# Parasitic diseases of equids in Iran (1931–2020): a literature review

**DOI:** 10.1186/s13071-020-04472-w

**Published:** 2020-11-19

**Authors:** Alireza Sazmand, Aliasghar Bahari, Sareh Papi, Domenico Otranto

**Affiliations:** 1 Department of Pathobiology, Faculty of Veterinary Science, Bu-Ali Sina University, Hamedan, 6517658978 Iran; 2 Department of Clinical Sciences, Faculty of Veterinary Science, Bu-Ali Sina University, Hamedan, 6517658978 Iran; 3 Department of Veterinary Medicine, University of Bari Aldo Moro, Str. prov. per Casamassima km 3, 70010 Valenzano, Bari, Italy

**Keywords:** Donkey, *Equus*, Horse, Iran, Mule, Parasite, Review

## Abstract

Parasitic infections can cause many respiratory, digestive and other diseases and contribute to some performance conditions in equids. However, knowledge on the biodiversity of parasites of equids in Iran is still limited. The present review covers all the information about parasitic diseases of horses, donkeys, mules and wild asses in Iran published as articles in Iranian and international journals, dissertations and congress papers from 1931 to July 2020. Parasites so far described in Iranian equids include species of 9 genera of the Protozoa (*Trypanosoma*, *Giardia*, *Eimeria*, *Klossiella*, *Cryptosporidium*, *Toxoplasma*, *Neospora*, *Theileria* and *Babesia*), 50 helminth species from the digestive system (i.e., 2 trematodes, 3 cestodes and 37 nematodes) and from other organs (i.e., *Schistosoma turkestanica*, *Echinococcus granulosus*, *Dictyocaulus arnfieldi*, *Parafilaria multipapillosa*, *Setaria equina* and 3 *Onchocerca* spp.). Furthermore, 16 species of hard ticks, 3 mite species causing mange, 2 lice species, and larvae of 4 *Gastrophilus* species and *Hippobosca equina* have been reported from equids in Iran. Archeoparasitological findings in coprolites of equids include *Fasciola hepatica*, *Oxyuris equi*, *Anoplocephala* spp*.* and intestinal strongyles. Parasitic diseases are important issues in terms of animal welfare, economics and public health; however, parasites and parasitic diseases of equines have not received adequate attention compared with ruminants and camels in Iran. The present review highlights the knowledge gaps related to equines about the presence, species, genotypes and subtypes of *Neospora hughesi*, *Sarcocystis *spp., *Trichinella* spp., *Cryptosporidium* spp., *Giardia duodenalis*, *Blastocystis* and microsporidia. Identification of ticks vectoring pathogenic parasites, bacteria and viruses has received little attention, too. The efficacy of common horse wormers also needs to be evaluated systematically.
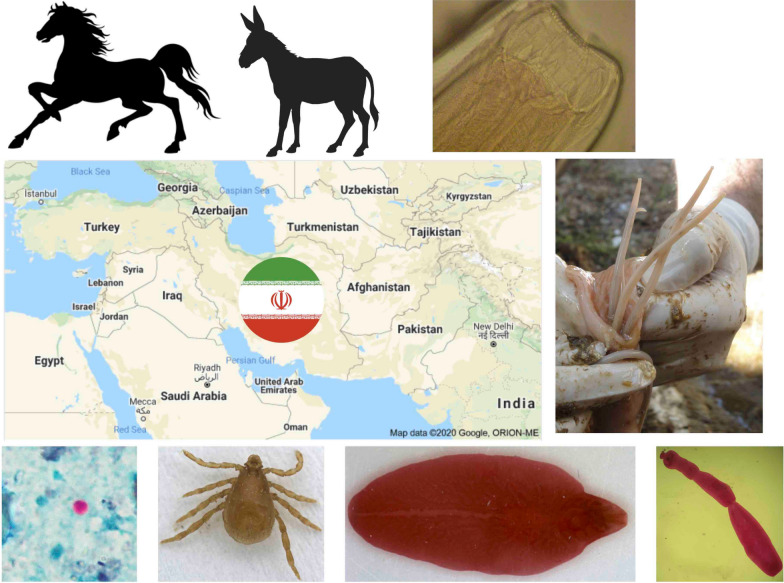

## Background

Horses were tamed and brought to Iran by the Aryans around 3000 years ago [1, 2]. In ancient Iran, the institutions for the treatment and care of animals and humans are similar, and sometimes the same person was responsible for treating both humans and animals. Ancient Iranians considered horses the most important animals, and ruminants were less so [2]. The great impact of horses was related to their use in guarding frontiers and in conquests [3]. Several infectious diseases were known to early veterinarians in Iran [2]. In his comprehensive book on horse medicine (*al-Furusiyah wa-al-Baytariyah*, in English: Equestrian and Veterinary Services), Ibn Akhi Hozam (died circa 842 A.D.), one of the most authoritative figures in Iranian veterinary literature, described the characteristics, habits and diseases of horses as well as treatment methods with a focus on mange mite infestation and itching in horses and its transmission to humans. Another scientist, Ali ibn Dawud (died circa 1363), described gastrointestinal helminthiases of horses in his book *Al-aqwal al-Kafiyah wa-al-Fusul al-Shafiyah fī al-Khayl* (in English: Enough Words and Treatment Classes in Horses) [4].

Modern veterinary knowledge found its way into Iran during the 1850s via European veterinarians who were mainly in charge of royal stables and military services [5]. However, the first record of veterinary parasitology activities in Iran is the 1931 report by Carpentier who diagnosed *Trypanosoma evansi* in the blood of sick horses in southern Iran [6]. Since the 1930s, Iranian veterinarians have been trained in faculties of veterinary medicine in Iran and have conducted modern veterinary parasitology research.

There is no updated official report on the population of equines in Iran; however, according to the Equestrian Federation of Iran, about 25,000 certified horses of different breeds such as Arabian, Turkmen, Thoroughbred, Darehshori, Kurd and Caspian are distributed throughout the country (H. Katebi, personal communication). Although there are numerous donkeys, working horses and mules in Iran, no information about their populations could be obtained from the Ministry of Agriculture or from the Veterinary Organization. In this article, we review the published research on equine parasites in Iran from 1931 to July 2020. For this purpose we checked all available documents on each of the search terms which included a combination of Iran or Iranian (in Persian, English and French) with each of the generic names of the parasites of equids as mentioned in the reference book “Veterinary Parasitology” [7], the “Handbook of Equine Parasite Control” [8] and checklist of strongylid parasites [9]. The databases and search engines employed for the present literature review were those of PubMed (www.pubmed.gov), Google (www.google.com), Scientific Information Database of Iran (www.sid.ir), the collection of defended theses at all Iranian universities (https://irandoc.ac.ir/) and the collection of proceedings of Iranian scientific congresses on veterinary medicine, animal science and parasitology (https://www.civilica.com/). Valid names of the reported parasites in older literature were obtained from updated resources [9, 10]. The abstract of present literature review in Persian language is provided as an Additional file 1.

## Protozoan infections

### Trypanosomosis

*Trypanosoma evansi* is one of the most pathogenic and economically important protozoan parasites of horses. The disease called “surra” in the acute form is characterized by symptoms such as fluctuating fever, weakness, lethargy, anemia, severe weight loss, transient local or general cutaneous eruption, petechial hemorrhages on the eyelids, vulvar and vaginal mucosa, hemorrhages into the anterior chamber of the eye, abortion, alteration of locomotion and edema."Chronic manifestations include a progressive weight loss (described as “living skeletons”), loss of appetite and emaciation accompanied by jaundice and highly colored urine [11].

Infection with *T. evansi* has been reported in horses, donkeys and mules in different countries in Asia, Africa, South America and Europe [12]. *Trypanosoma evansi* is now considered as an emerging zoonotic parasite [13]. *Trypanosoma* was first reported in Iran in 1876 and was known to be fatal for horses [14]. After massive mortality of > 3000 horses in 1930 in the south of Iran, *T. evansi* was diagnosed in the blood of diseased animals [6]. In 1935, an outbreak of surra occurred in camels in the surroundings of Tehran [15]. Experimental infection of horse and donkey with *T. evansi* isolated from an infected camel caused disease after an incubation period of 6 days but the animals could survive after 40 days by the end of the experiment [15, 16]. Although infection rates of up to 19.47% for *Trypanosoma* spp. infections have been reported in camels from different regions of the country [17], there are few reports on *T. evansi* infection in equids. *Trypanosoma evansi* was diagnosed in two outbreaks of surra in mules in Dezfoul in 1961 and in horses in Kharameh in 1993 [18]. Association of *T. evansi* infection in a mare with abortion in Shiraz has also been documented [19]. Since Iran does not lie within the tsetse belt, trypomastigotes in animals have usually been assigned to *T. evansi* according to their morphological and morphometric features upon microscopic examination. *Trypanosoma* spp. were not detected in the only PCR-based study on 116 horses, donkeys and mules from six geographical regions in Iran [20]. Although no study has been conducted to define vectors of *T. evansi* in the country, reports of natural infections with *T. evansi* in camels [21], dogs [22] and water buffalo [23] with no history of travel show that non-cyclic transmission occurs.

*Trypanosoma equiperdum* is another important veterinary trypanosome infecting equids. Dourine is a disease caused by this species and is endemic in Africa and Asia; it is also found in the Middle East, southeastern Europe and South America. *Trypanosoma equiperdum* is the only trypanosome with transmission occurring during copulation of horses. However, experimental infections inoculating parasites via the intravenous or intraperitoneal route indicate that mechanical transmission by blood-feeding flies cannot be excluded as a possible route [24]. The infection presents with typical edema of the genital organs as well as weakness, emaciation, urethral discharge, characteristic plaques in the skin and neurological symptoms such as lack of coordination of the hind legs. Dourine in horses is generally fatal without treatment but is usually subclinical in donkeys and mules [25]. In Iran, dourine and its chemical treatment have been reported by veterinarians in southern and western areas since the 1930s. As in the 1940s in Iran, suramin (Naganol), arsphenamine (Salvarsan), Neosalvarsan and other common medications were extremely expensive, new treatment protocols with oral arsenic and orpiment accompanied by intravenous injection of tamsulosin or oral arsenic accompanied by intra-arterial injection of Suramin were developed [26]. *Trypanosoma equiperdum* has been isolated only once in Iran [27]. In one study on 119 equids (75 horses and 44 donkeys and mules) in Gonbad region, north of Iran, *T. equiperdum* was not detected in blood smears, but using mercuric chloride and formol gel tests 48% of horses and 77.27% of donkeys and mules were positive for the infection [28]. In Fars Province, 16 equids (10 stallions, 5 mares and 1 male donkey) were suspected to be infected with *T. equiperdum* according to their clinical characteristics such as edema in the genital organs and paraphimosis in stallions and cutaneous plaques with skin thicknesses on the neck and chest in mares, though no *Trypanosoma* was observed in clinical samples [29]. In a single case report, *T. equiperdum* was detected microscopically in a genitalia wash of a stallion from the southeast of Iran [30].

### Giardiosis

There are very few data on *Giardia duodenalis* in horses although the parasite is commonly found in feces of asymptomatic animals. Although uncommon, giardiosis in horses has been found to be associated with diarrhea, a poor hair coat, ill thrift and weight loss. Assemblages A, B and E have been identified in horses [31]. All of these assemblages are pathogenic for humans so horses could represent a reservoir of *G. duodenalis* with the potential to cause disease in humans through direct contact or by contamination of food and/or water supplies.

There are only two reports on *Giardia* infection in horses in Iran. In one study from Ahvaz (southwestern Iran), trichrome-stained fecal smears of 100 racing horses of different ages were studied microscopically and 40% were found infected with cysts of *Giardia* [32]. In another study conducted in the same region, 35.7% of the fecal samples of Arabian horses (*n* = 42) were molecularly positive for *G. duodenalis*. Assemblages E and AI were the most prevalent [33].

### Coccidiosis (*Eimeria lueckarti* and *Klossiella equi*)

Until recently, there was confusion concerning the etiological identity of *Eimeria* species in equids. A comprehensive examination of samples and literature in 2018 led to the recognition of *E. leuckarti* as the only valid species of *Eimeria* in equids, which has been consistently found in numerous surveys worldwide [34]. *Eimeria leuckarti* infects the horse (*Equus caballus*), donkey (*Equus asinus*), mule (*Equus mulus*), Asian wild ass (*Equus hemionus*), Mountain zebra (*Equus zebra*) and Grant’s zebra (*Equus quagga*). Infections are more common in foals than in adult animals. Foals can acquire infection on the day of birth, probably from the contaminated environment rather than from oocysts excreted by their mares. Most infections are considered coincidental or without clinical relevance, although enteritis has been reported in a few cases [34]. In Iran, there are few reports on eimeriosis in equids with infection rates of 0.5–57.14% in horses [35–39] and 7.7% in donkeys [36]. As many other researchers who performed coproscopy did not report *Eimeria* oocysts in fecal examinations, it seems that eimeriosis is not common in the country considering that equines in Iran are not treated with coccidiostatic and coccidiocidal compounds.

There is also one report of *Klossiella equi*, a rare coccidian parasite of the renal parenchyma of equids with no clinical signs in the kidneys associated with this parasite [40]. In 2011, different developmental stages of the parasite were observed in histopathological study of renal sections of a 10-year-old donkey (*Equus asinus asinus*) [41].

### Cryptosporidiosis

*Cryptosporidium* spp., gregarines that infect a wide range of vertebrates, are the causative agents of zoonotic infections associated with food- and waterborne outbreaks [42]. *Cryptosporidium* spp. have a fecal-oral transmission route and induce a self-limiting disease in immunocompetent individuals, but it may cause a debilitating infection with typical aqueous diarrhea and weight loss in infants, young animals and immunocompromised individuals [43]. In equines, clinical manifestations are rare; however, generally the pathogenicity of *Cryptosporidium* spp. depends on both the genetic background and immune status of the animals and the virulence of the specific genotypes and subtypes involved [44]. In horses and donkeys *Cryptosporidium* horse genotype, *C. parvum* and *C. hominis* are responsible for > 90% of infections. *Cryptosporidium andersoni* and *C. muris* have been reported in at least five cases while *C. tyzzeri*, *C. felis*, *C. erinacei*, *C. proliferans* and *C. ryanae* have each been reported in fewer than five animals [45–47]. All of these species and genotypes except *C. proliferans* have been reported in humans [48]. In Iran infection rates of 2.0–26.7% in horses and mules of different regions have been reported (Table 1). There are also two reports of possible transmission of *Cryptosporidium* to humans related with them [49, 50]. However, all of the studies on equine cryptosporidiosis were based on microscopic examination of Ziehl-Neelsen-stained fecal smears.

**Table 1.** Prevalence rates of *Cryptosporidium* spp. in equids in Iran

Host	Province	No. of examined animals	Percentage of positive animals	References and year^a^
Horse	Golestan	500	8	[51] 1993
Horse	East Azerbaijan	500	5.8	[49] 1996
Horse	Khorasan Razavi	300	26.7	[50] 2002
Horse	West Azerbaijan	221	15.8	[52] 2007
Horse	East Azerbaijan	50	6 in winter	[53] 2008
			2 in autumn	
Horse	Tehran	200	25	[54] 2010
Horse	Golestan	400	5.4	[55] 2010
Horse	West Azerbaijan	70	10.6	[56] 2012
Mule		72	12.5	
Horse	Hamedan	158	12.6	[57] 2012
Horse	Khuzestan	100	18	[58] 2013

^a^Year of publication

### Toxoplasmosis

*Toxoplasma gondii* is an obligate intracellular protozoan parasite that can infect an exceptionally wide range of warm- and cold-blooded animals as well as humans making it one of the most widespread parasites on earth [59]. Approximately 30% of the world’s population is infected with this cosmopolitan food- and water-borne parasite [60].

In equids worldwide, *T. gondii* has been detected by both indirect (serology) and direct (PCR, mouse bioassay) tests although there is no confirmed report of clinical toxoplasmosis in horses suggesting that they might be resistant to toxoplasmosis [61]. Several epizootiological studies have been conducted for the detection of anti-*Toxoplasma* antibodies in blood serum of horses and donkeys from Iran (Table 2). There is also one research article on detection of *T. gondii* in blood samples by PCR-RFLP in Urmia Province. In that work, 2 of 126 horses (1.6%) tested positive [62]. Accordingly, eating raw horse meat may expose consumers to *T. gondii* [63] with severe cases of human toxoplasmosis reported in France due to the consumption of imported South American and North American horsemeat contaminated with highly pathogenic strains of the parasite [64]. However, as meat of equids is not consumed in Iran because of cultural and religious beliefs, horses and donkeys might not play a significant role in the epidemiology of human infections in the country. Furthermore, it has been shown that drinking raw milk of livestock can pose a risk to humans [65]. As consumption of milk of horses and donkeys has recently become popular in the country, proper thermal treatment of milk should receive more consideration.

**Table 2.** Seroprevalence of *T. gondii* antibodies in blood serum of equids in Iran

Host	Province	Diagnosis	No. of examined animals	Percentage of positive animals	References and year^a^
Horse	Fars	DT	110	4.5	[66] 1991
Horse	Qazvin	MAT	52	71.2	[67] 2010
Horse	West Azerbaijan	MAT	26	11.5	[68] 2011
Horse	Fars	MAT	35	40.0	[69] 2012
Horse	Fars	MAT	35	40.0	[70] 2013
Horse	North Khorasan	IFAT	100	41.0	[71] 2013
Horse	Hamedan	MAT	120	13.3	[72] 2014
Horse	Khuzestan	MAT	235	48.5	[73] 2015
Horse	Kerman and Yazd	LAT	163	43.6	[74] 2017
Donkey	Hamedan	MAT	100	47.0	[75] 2015

*NS* not stated, *DT* dye test, *MAT* modified agglutination test, *IFAT* immunofluorescent antibody test, *LAT* latex agglutination test

^a^Year of publication

### Neosporosis

Horses are known to be intermediate hosts of *Neospora hughesi*, which seems to represent a species different from *N. caninum*. *Neospora hughesi* causes myeloencephalitis, forming tissue cysts smaller than those of *N. caninum* with thinner cyst walls and smaller bradyzoites [76]. It is, however, not clear whether *N. hughesi* is the sole species of *Neospora* that infects horses. Despite the antigenic and molecular differences between the two species [77], *N. caninum* tachyzoites were used as antigens in all serological prevalence studies on horses and donkeys in Iran. In these studies, seroprevalences of 20.0–40.8% in horses [73, 78–81] and 52% in the only study on donkeys were reported [81].

### Piroplasmosis (*Theileria equi* and *Babesia caballi*)

Equine piroplasmosis, caused by two hemoprotozoan apicomplexan parasites (*Theileria equi* and *Babesia caballi*), is a tick-borne disease of horses, mules, donkeys and zebras that has also been reported in dogs and dromedary camels, therefore raising doubts about piroplasm host specificity [82, 83]. At least 33 ixodid species within the genera *Hyalomma*, *Dermacentor*, *Rhipicephalus*, *Ixodes*, *Amblyomma* and *Haemaphysalis* have been implicated as competent vectors for *B. caballi*, *T. equi* or both [84]. In endemic areas, there are no overt clinical signs of infection but clinical disease can manifest in different forms as subacute or chronic [85]. Acute disease may also occur and is characterized by fever (temperature > 40 °C), malaise, reduced appetite, anorexia, constipation followed by diarrhea, tachycardia, tachypnea, petechiae, splenomegaly, thrombocytopenia and hemolytic anemia leading to hemoglobinuria and icterus [86].

Piroplasmosis was first reported in the Iranian literature in the 1930s when *B. caballi* was diagnosed in the blood smears of Hungarian mares imported to Iran [6]. It was not discovered whether the infection of these horses occurred in Iran or outside the country [87]. In the 1940s, microscopic and serological diagnosis of both *B. caballi* and *T. equi* (referred to as *Piroplasma caballi* and *Nuttallia equi*) were performed, and infected horses were treated with Gonacrine^®^ (3,6-diamino-10-methylacridinium chloride; SPECIA, Paris, France), Trypaflavin^®^ (acriflavine hydrochloride; Bayer, Leverkusen, Germany) and Acaprin^®^ (quinuronium sulfate; Bayer, Leverkusen, Germany) [88, 89]. For almost 50 years there has been no report of piroplasmosis, but since 1992, and upon diagnosis of a horse with *T. equi* [90], research and reports have begun again. Although clinical disease with *B. caballi* and *T. equi* has been documented sporadically [91–95], epizootological studies (Table 3) show that both theileriosis and babesiosis are present all over the country, and infected horses, donkeys and mules are subclinically affected. Not much is known about ticks vectoring equine piroplasms but DNA of *T. equi* has been found in *Hyalomma excavatum* and *Rhipicephalus bursa* ticks collected from infected horses [96].

**Table 3.** Prevalence of *Theileria equi* and *Babesia caballi* in equids in Iran

Host	Area of study	Diagnosis	No. of examined animals	Percentage of positive animals	Pathogen	References and year^a^
Horse	Mianeh county	Microscopy	122	4.10	*T. equi*	[97] 2010
Donkey			178	0		
Horse	North Khorasan province	IFAT	100	48.0	*T. equi*	[96] 2014
				2.0	*B. caballi*	
				3.0	Mixed	
		cPCR+ seq		0	*T. equi*	
				45.0	*B. caballi*	
				0	Mixed	
Horse	Urmia county	cPCR	240	6.25	*T. equi*	[98] 2014
				2.80	*B. caballi*	
				1.66	Mixed	
Horse	Yazd province	cPCR	105	22.86	*T. equi*	[99] 2014
Horse	Khuzestan province	cPCR	165	28.50	*T. equi*	[100] 2014
Horse	Tabriz county	cPCR	95	8.42	*T. equi*	[101] 2014
				2.10	*B. caballi*	
Horse	Ahvaz city	cPCR	45	46.67	*T. equi*	[102] 2015
Donkey	North Khorasan province	cPCR+ seq	160	50.94	*T. equi*	[103] 2015
				0	*B. caballi*	
Horse and mule	Piranshahr county	cPCR+ seq	31 (24 horses, 7 mules)	96.77	*T. equi*	[104] 2016
				0	*B. caballi*	
Horse	Ardabil province	Real-time PCR	92	4.35	*T. equi*	[105] 2016
				9.78	*B. caballi*	
Horse	Kerman province	Microscopy	246	0.81	*T. equi*	[106] 2017
Horse	Kordestan province	cPCR+ seq	186	0.54	*T. equi*	[107] 2017
				0	*B. caballi*	
Horse	Esfahan county	cPCR	53	11.32	*T. equi*	[108] 2017
				9.43	*B. caballi*	
	Shahrekord county		37	10.81	*T. equi*	
				13.51	*B. caballi*	
Donkey	Kordestan province	cPCR	232	0	*T. equi*	[109] 2018
				0	*B. caballi*	
Horse	West Azerbaijan province	Microscopy	126	2.17	*T. equi*	[110] 2018
		cPCR		27.78	*T. equi*	
Horse	Lorestan province	cPCR	165	15.75	*T. equi*	[111] 2018
				8.48	*B. caballi*	
				5.45	Mixed	
Horse	Fars province	cPCR	133	2.55	*T. equi*	[112] 2020
				0	*B. caballi*	
Donkey	Urmia county	Microscopy	200	3.50	*T. equi*	[113] 2020
				0	*B. caballi*	
		cPCR		5.50	*T. equi*	
				1.50	*B. caballi*	

*IFAT* immunofluorescent antibody test, *cPCR* conventional PCR, *seq* nucleotide sequencing

^a^Year of publication

## Helminthoses

### Nematodoses

#### Infections with strongylids (Nematoda: Strongylidae)

The nematode parasites of horses belong to 7 suborders, 12 families, 29 genera and 83 species. The majority (19 of 29 genera and 64 of 83 species) are members of the Strongylidae family, which includes the most common and pathogenic nematode parasites of horses [9]. Migratory strongylids (*Strongylus vulgaris*, *Strongylus edentatus* and *Strongylus equinus* commonly named “large strongyles”) occur in the large intestine. Clinically, these are the most important of the equine parasites with *S. vulgaris* considered a major threat to equine health. Large strongyles might cause severe pathological consequences and clinical signs, which differ depending on the species. On the other hand, non-migratory strongylids (commonly named “small strongyles”) include cyathostomins and non-migratory strongyline species such as *Triodontophorus* spp., *Craterostomum* spp. and *Oesophagodontus* spp., which are very common nematode parasites of equids. Non-migratory strongylids are considered much less pathogenic; however, many of these worms may damage the intestinal mucosa and result in emaciation and diarrhea, which is sometimes accompanied by colic, weight loss, fever and even death [8, 9, 114].

In Iran, infection of equids with large and small strongyles has commonly been reported in horses, donkeys and mules following coproscopic examinations or fecal culture under general terms such as “*Strongylus* spp. eggs,” *Strongylus* spp. larvae,” “cyathostomins eggs” and “cyathostomins larvae.” Infection rates between 4.4 and 69.2% in horses [35–37, 114–127], 65.4 and 96.4% in four studies on donkeys [36, 117, 128, 129] and 80% in the only study on mules [117] have been reported.

Based on necropsies, 27 species of strongylids from 10 genera have been recorded in Iran. In some instances, the species names were corrected according to the checklist of Lichtenfels et al. [9]. Based on our intensive searches, the strongylid fauna of horses and donkeys in Iran consists of members of the genera *Strongylus* (*n* = 3), *Oesophagodontus* (*n* = 1), *Triodontophorus* (*n* = 3), *Cyathostomum* (*n* = 4), *Coronocyclus* (*n* = 3), *Cylicodontophorus* (*n* = 1), *Cylicocyclus* (*n* = 5), *Cylicostephanus* (*n* = 5), *Gyalocephalus* (*n* = 1) and *Poteriostomum* (*n* = 1) (Table 4).

**Table 4:** Strongylinae and cyathostominae species recovered from horses and donkeys in Iran

Species	Host	References
*Strongylus edentatus* (Looss, 1900)	H	[35, 115, 130]
	D	[128, 131]
*Strongylus equinus* (Müller, 1780)	H	[35, 115, 130]
	D	[128, 131]
*Strongylus vulgaris* (Looss, 1900)	H	[35, 115, 130, 132, 133]
	D	[117, 128, 131, 134]
*Oesophagodontus robustus* (Giles, 1892)	H	[115, 130]
*Triodontophorus serratus* (Looss, 1900)	H	[115, 135, 136]
	D	[131, 135, 137]
*Triodontophorus brevicauda* (Boulenger, 1916)	H	[135, 136]
	D	[135]
*Triodontophorus tenuicollis* (Boulenger, 1916)	H	[135]
	D	[135]
*Triodontophorus* spp.	H	[130]
*Cyathostomum tetracanthum* (Mehlis, 1831) also reported as *Trichonema tetracantum*, *Trichonema aegyptiacum*	H	[115, 135, 138]
	D	[131, 135]
*Cyathostomum alveatum* (Looss, 1900)	H	[135]
	D	[131, 135]
*Cyathostomum catinatum* (Looss, 1900)	H	[135, 136, 138]
	D	[135]
	M	[139]
*Cyathostomum pateratum* (Yorke & Macfie, 1919)	H	[135, 138]
	D	[135, 140]
*Coronocyclus coronatus* (Looss, 1900) also reported as *Cyathostomum coronatum*	H	[135, 136, 138]
	D	[131, 135]
	M	[139]
*Coronocyclus labiatus* (Looss, 1902) also reported as *Cyathostomum labiatum*	H	[135, 136, 138]
	D	[131, 135]
	M	[139]
*Coronocyclus labratus* (Looss, 1900) also reported as *Cyathostomum labratum*	H	[135, 138]
	D	[131, 135]
*Cylicodontophorus bicoronatus* (Looss, 1900)	H	[135]
	D	[135]
	M	[139]
*Cylicodontophorus* spp.	H	[115]
*Cylicocyclus radiatus* (Looss, 1910)	H	[135, 138]
	D	[131, 135]
*Cylicocyclus auriculatus* (Looss, 1900)	H	[135, 138]
	D	[131, 135]
*Cylicocyclus elongatus* (Looss, 1900)	H	[135, 138]
	D	[135, 140]
*Cylicocyclus insigne* (Boulenger, 1917)	H	[135]
	D	[135]
*Cylicocyclus nassatus* (Looss, 1900) also reported as *Cylicocyclus nassatum*	H	[115, 135, 138]
	D	[131, 135]
*Cylicostephanus minutus* (Yorke & Macfie, 1918)	H	[135, 138]
	M	[139]
*Cylicostephanus calicatus* (Looss, 1900)	H	[135, 136, 138]
	M	[139]
*Cylicostephanus goldi* (Boulenger, 1917) also reported as *Trichonema parvibursatum*	H	[115, 135, 138]
	D	[131, 135]
	M	[139]
*Cylicostephanus hybridus* (Kotlan, 1920) also reported as *Trichonema hybridum*	H	[115]
*Cylicostephanus longibursatus* (Yorke & Macfie, 1918) also reported as *Trichonema longibursatum*	H	[115, 135]
	D	[131, 135]
	M	[139]
*Gyalocephalus capitatus* (Looss, 1900)	H	[115, 135, 138]
	D	[128]
	M	[139]
*Poteriostomum imparidentatum*	M	[139]
*Poteriostomum* spp.	H	[115]
	D	[128]

*H* horse, *D* donkey, *M* mule

#### Parascariosis (Parascaris equorum and Parascaris univalens)

*Parascaris* spp. reside in the small intestine of equids and are one of the largest known parasitic nematode species measuring up to 50 cm in length at the adult stage. Following ingestion of the third-stage larva (L3) within the egg, larvae are released and penetrate the small intestinal wall to begin a somatic migration via the bloodstream through the liver, heart and lungs. Then, the larvae migrate proximally in the pulmonary tree or are coughed up into the pharynx where they are swallowed and return to the stomach and small intestine, growing progressively to adult ascarids [8, 114].

The tradition in veterinary parasitology has been to refer to one equine ascarid species, *Parascaris equorum*. However, the published literature contains scant mention of a cryptic ascarid species infecting horses, named *Parascaris univalens* [141, 142], which is the dominating species infecting horses in Sweden, Switzerland and the USA, and possibly globally [143]. A sequence of the mitochondrial cytochrome *c* oxidase subunit 2 (*cox*2) gene (GenBank: MG676884) from Iranian isolates shows > 99% identity with sequences for both *P. equorum* and *P. univalens* available on GenBank [144]. As *P. equorum* and *P. univalens* are notoriously difficult to distinguish morphologically and their mitochondrial DNA genomes are very similar [145], cytological analysis of chromosome organization and the phenomenon of “chromatin diminution” [*P. univalens* (2*n* = 2) and *P. equorum* (2*n* = 4)] is the only established technique for differentiating these two species [146]. Since none of the reports of *P. equorum* in Iran were accompanied by karyotyping, we use *Parascaris* spp. in this article.

Infection of horses with *Parascaris* spp. is a common infection in equids, and prevalences of 12.2–40.0% in horses [35, 36, 115, 116, 118, 121–126, 133, 136, 147, 148] and 3.8–20.0% in three studies on donkeys [36, 117, 129] have been reported. Karyotyping of *Parascaris* spp. of equids in Iran will shed light on the distribution of *P. univalens* and *P. equorum* in the country.

#### Pinworm infections (Oxyuris equi and Probstmayria vivipara)

Nematodes of the Oxyuroidea, or pinworms, reside in the posterior alimentary tract. *Oxyuris equi*, the common pinworm of the equids, is rarely considered a major threat to equine health, but heavy infections may result in fatigue, decreased performance and loss of body condition. However, females of *O. equi* protrude from the anus and deposit eggs in a sticky film in the perineal area that becomes irritating to the host when the proteinaceous fluid dries. Consequently, horses rub their tail heads and rumps against fixed objects, causing local damage to the skin, haircoat and tail [8, 114]. *Probstmayria vivipara*, a less well-known pinworm species of equids, can complete its life cycle without leaving the host. *Probstmayria vivipara* is only detectable microscopically and is not known to be pathogenic to the horse even though populations may number in the hundreds of thousands [8]. In Iran, eggs and adults of *O. equi* have been reported (mostly in fecal egg count) in horses [35, 37, 115, 116, 118, 122, 124, 136, 148] and donkeys [36, 131, 148]. *Probstmayria vivipara*, however, has only been reported in necropsied donkeys with > 500,000 worms collected from a single animal [131, 149].

#### Stomach worm infections (Habronema spp. and Draschia spp.)

Equid habronemosis is a widespread parasitic disease caused by three species of spirurid nematodes that reside as adults in the stomach, *Habronema microstoma* (the most common), *Habronema muscae* and *Draschia megastoma*. Habronemosis causes mild to severe clinical symptoms, i.e., gastric, cutaneous, mucocutaneous and pulmonary diseases, depending on the parasite’s stage of development and on localization. Their life cycle requires an intermediate host, represented by dung-inhabiting secretophagous or hematophagous muscid flies [150]. As the egg stages of *Habronema* and *Draschia* are quite small and might not survive the flotation process, the diagnosis is mainly based on necropsy findings. In Iran, since the first report of *H. muscae* in horses in 1966 [151], all three species of stomach worms have been reported from horses as either single or multiple infections [115, 136, 152–154]. Examination of 45 slaughtered donkeys in a study showed very high infection rates with *H. muscae* and *H. microstoma* (80% and 66.6%) while 13.3% of donkeys were infected with *D. megastoma* [131]. Ocular habronemosis in one horse with conjunctivitis and lacrimation [155] and also summer sore cases [156, 157] have been reported. Gastric infection of Persian onager (*Equus hemionus onager*) with *H. muscae* has been documented, too [158].

#### Trichurosis

Equids are not common hosts for *Trichuris* species, but there are a few reports of detection of parasite eggs from Iran, specifically in fecal examinations of two horses, two donkeys and one mule [117]. Infection of horses, donkeys and mules has also been reported from Turkey [159, 160]. Almost 40 years ago adult male and female *T. suis* were isolated from the cecum and colon of a dead horse [161]. However, there is no other report on finding adult whipworm nematodes in equids.

#### Lungworm infections

*Dictyocaulus arnfieldi* infects the respiratory tract of equids worldwide [8]. Donkeys are more suitable hosts of *D. arnfieldi* than horses as donkeys can tolerate large numbers of parasites with few clinical signs of overt respiratory disease [162]. In a comparative study, the prevalence of the infection was 65% in donkeys, 22.72% in mules and 4.54% in horses, and the mean intensity was 34.3 worms in donkeys, 36.5 in mules and 2.0 in horses [163]. In Iran, eggs and larvae of *D*. *arnfieldi* have been examined in feces of horses, donkeys and mules [37, 164, 165] but few scientific data are available.

#### Threadworm infections

Female *Strongyloides westeri* nematodes parasitize the small intestine of equids. The infection is mainly observed in suckling foals, but older horses are occasionally found infected as well. Infection occurs via skin penetration by third-stage larvae, ingestion of third-stage larvae from a contaminated environment or lactogenic transmission from the mare. Equine strongyloidosis is recognized as a rare cause of disease [166]. Adult *S*. *westeri* nematodes in horses [115] and eggs or larvae in feces of horses [125, 167] and donkeys have been reported in Iran [129, 148].

#### Trichostrongylosis

*Trichostrongylus axei* is the only gastrointestinal nematode that equines share with ruminants via cross-infection. This parasite occurs in the abomasum of sheep, cattle, goats and camels in Iran [167–170]. Infection of human patients with *T. axei* has also been reported [171]. Adult nematodes have been isolated from horses [115] and donkeys [128, 131]. There are also some reports on detection of eggs of *T. axei* in coproscopic examinations in horses [125, 172], donkeys and mules [172]. However, eggs of *T. axei* are morphologically indistinguishable from those of the other intestinal strongyles, and differentiation requires fecal culture [114]. As coproculture was not performed in the latter studies, the results remain dubious.

#### Filarioidean infections (Parafilaria spp., Onchocerca spp. and Setaria spp.)

Adult *Parafilaria multipapillosa* occurs in subcutaneous and intermuscular connective tissue of equines. Nodules form in the overlying skin, and the nodules may rupture and bleed or leak tissue fluids [8]. In Iran, microfilariae of *P. multipapillosa* have been reported in peripheral blood samples [173–175] and embryonated eggs and/or microfilariae from hemorrhagic discharges of skin nodules [176] of horses and donkeys. The enzootic areas for *P. multipapillosa* are the Caspian littoral, steppes and forest steppes with temperate-wet climate and altitudes of up to 1500 m [176].

Regarding the *Onchocerca* species, infection of equids with four species of *Onchocerca*, i.e., *O. reticulata*, *O. cervicalis*, *O. raillieti* and *O. boehmi* (syn. *Elaeophora boehmi*), have been reported [177]. Adult nematodes are found deep in the connective tissues such as in the nuchal ligament area and the distal limbs [8]. In Iran, microfilariae of *O. reticulata*, *O. cervicalis* and *O. boehmi* have been examined in peripheral blood samples of horses and donkeys [173, 175, 178]. However, so far there is no report on *Onchocerca* nodules in equids in the country.

*Setaria equina* is a filarioid nematode that lives free in the abdominal cavity of equids. Equine setariosis is considered non-pathogenic in most cases although serious pathogenic effects could occur when they reside in unusual habitats such as the eye, brain, spinal medulla and testicles of horses [179]. In Iran, microfilariae of *S*. *equina* have been examined in peripheral blood [175, 178], and adults have been isolated from the abdominal cavity of donkeys [131], in the testicles of one horse [180], in the cecum and colon of two horses [130] and eyes of two horses [181]. There is also one report of subconjunctival setariosis due to *S. equina* in a 15-year-old girl in Tabriz, a city in the northwest of the country [182].

#### Eyeworm infections

Horses are the definitive hosts of *Thelazia lacrymalis* and *T. rhodesi* transmitted by many species of muscid face flies according to the geographical distribution [183]. Adult worms usually reside in the conjunctival cul-de-sac, beneath the nictitating membrane, in excretory ducts of the Harderian gland, in the ducts of the lacrimal glands or rarely free in the conjunctiva [184]. Pathologies range from no gross lesions to mild conjunctivitis, photophobia and lacrimation up to severe lesions such as keratitis and in some chronic cases corneal ulceration [185]. Eyeworm infections of equids have not received any attention in Iran. In an old document, the author mentioned one case of *T. rhodesi* infection in a horse from Babol, northern Iran [186]. However, in a study on ophthalmic diseases of 901 horses in Tehran, no eyeworms were reported [187]. In a report from 2014, eyeworms of one horse in the southeastern region of the country was diagnosed with *T. lacrymalis* [188]. As both species *T. lacrymalis* and *T. rhodesi* are endemic in several regions of Iran [189, 190], equine thelaziosis seem to be underdiagnosed.

#### Gullet worm infections

*Gongylonema* spp., also known as gullet worms, are globally distributed parasitic nematodes that reside in the upper digestive tract of a wide range of domestic and wild mammals [191]. Although human infections with this nematode are rare, over 60 cases have been reported worldwide [192]. In Iran, *Gongylonema* spp. have been diagnosed in cattle, sheep, buffalo, goats, camels, wild boars and a human patient [170, 193, 194] and are reported as *G. pulchrum* by tradition. In the only report from equines of Iran, *Gongylonema* spp. was diagnosed in the esophagus and stomach of a donkey [195]. Gongylonemosis has long been known to occur in horses and donkeys [196]. However, recent separation of *G. nepalensis* from *G*. *pulchrum* (which is almost identical in morphology except for distinctly shorter left spicules) in addition to reports of infection in buffaloes, cattle, sheep, goats, wild European mouflons and a red fox [191] would suggest that equids can also be hosts for *G. nepalensis*.

### Cestodosis

#### Tapeworm infections (Anoplocephala perfoliata and Anoplocephala magna)

Anoplocephalid cestodes occur worldwide and are potential causes of various forms of colic. These tapeworms utilize intermediate hosts, comprising numerous species of oribatid mites ingested accidentally by horses during grazing. Three species of cestodes, i.e., *Anoplocephala perfoliata*, *A. magna* and *Anoplocephaloides mamillana*, are known to infect equids; *A. perfoliata* is by far the most prevalent [197]. In Iran, the two *Anoplocephala* species have been reported in horses [115, 130, 198] and donkeys [131, 199]. Eggs of *Anoplocephala* spp. have also been detected in feces of horses [35, 37, 125] though identification was not performed except for one study reporting the eggs as *A. perfoliata* [200].

#### Hydatidosis (Echinococcus spp.)

In equines cystic echinococcosis (CE) is generally a rare finding, mostly incidentally diagnosed at slaughter or postmortem examination. The hydatid cysts commonly develop in the liver and lungs. The cysts have a reported longevity of several years and rarely cause severe clinical symptoms [201]. Cases of equine cystic hydatidosis have been reported from Europe, the Middle East, South and East Africa, North America and Southeast Asia [202]. In many of these cases, the identity of the causative *Echinococcus* taxon was not confirmed [203]. It is assumed that *E. equinus* (horse strain/G4) is the only species that produces fertile cysts in equines whereas the recovery of small sterile cysts of *E. granulosus* in horses confirms that the horse is not an efficient host for this species [204]. *Echinococcus equinus* is probably not infective to humans [205]. Cystic echinococcosis is hyperendemic in Iran, and occurrence of *E. granulosus* (G1–G3), *E. ortleppi* (G5) and *E. intermedius* (G6/7) in dogs, humans and livestock, i.e., sheep, goats, cattle and camels, is extensively reported [206]. Regarding infection of equids, however, hydatidosis has not received adequate attention, and only three articles are available. In serodiagnosis of hydatidosis in horses, only 6 sera of 193 samples (3.11%) tested positive [207]. In donkeys, it can be concluded that both *E. granulosus* and *E. equinus* are present. In a study in 1987, the authors stated that hydatid cysts in the lungs of two donkeys did not have protoscolices [199] but in 2014 hydatid cysts in the liver of one infected animal harbored protoscolices with morphological characteristics consistent with previous descriptions in Switzerland and Jordan [208]. In the latter study, nucleotide sequences of a partial sequence of *cox*1 from donkey were similar to the corresponding sequence of *E. equinus* in GenBank [208]. Dogs, black-backed jackals (*Canis mesomelas*) and interestingly lions (*Panthera leo*) have been identified as definitive hosts of *E. equinus* [209]. So far, *E. equinus* has not been recorded in the canine in Iran [206].

### Trematodosis

#### Liver fluke infections (Fasciola spp. and Dicrocoelium spp.)

Horses and donkeys can acquire infection with both *Fasciola hepatica* and *F. gigantica* in their liver [210, 211]. Infected horses with liver flukes show clinical signs including poor performance, fatigue, diarrhea, inappetence and jaundice [212]. Very recently failure to establish infection of horses after oral challenge with metacercariae raised fundamental questions on the pathophysiology and epidemiology of equine fasciolosis [213]. In Iran, eggs of *Fasciola* spp. have been found in feces of horses with a prevalence of 3–50% [35, 136, 214, 215], and adult *F. hepatica* flukes have been isolated from bile ducts of donkeys [131, 199].

Dicrocoeliosis is caused by several *Dicrocoelium* spp. that live in the bile ducts and gall bladder of domestic and wild ruminants but occasionally affect other animals including horses and humans [216]. In Iran, eggs of *Dicrocoelium* spp. have been reported from 1 to 33.3% of horses [122, 125, 136]. Adult flukes have also been isolated from livers of 6.7% of donkeys in two studies [131, 199]. Equine hepatic *Dicrocoelium dendriticum* infection has also been reported from Azerbaijan, Turkey, Denmark, Nigeria, Switzerland and Canada [217, 218]. However, there is no information regarding the clinical effect of the lancet fluke in equids due to lack of experimental infections. Furthermore, although *D. dendriticum* is the most widespread liver fluke worldwide, special care must be taken for reporting *Dicrocoelium* spp. eggs in feces as eggs of *D. dendriticum*, *D. suppereri* and *D. hospes* are similar morphologically [219, 220].

#### Schistosomosis

Horses, donkeys and mules are susceptible to a wide range of schistosomes, e.g., *S. bovis*, *S. japonicum*, *S. indicum*, *S. nasale*, *S. spindale* and *Heterobilharzia americana* [220–224]. In Iran, where *S. turkestanica* is endemic, the infection has been reported from cattle, sheep, goats, buffaloes and camels in addition to causing cercarial dermatitis in humans [225, 226]. An article dated 1973 mentioned that one donkey in southwestern Khuzestan was found infected with a few *S*. *turkestanica* worms and concluded that donkeys were not important hosts for this parasite [227]. After almost 35 years of no report, eggs of *S*. *turkestanica* were detected in feces of two horses in northwestern Iran [125].

## Arthropod infections

### Tick infestation

Ticks play a vital role in the stable maintenance and natural transmission of several equine-infective tick-borne pathogens, including protozoa (e.g., *T. equi*, *B. caballi*) [84], bacteria (e.g., *Anaplasma phagocytophilum*, *Borrelia burgdorferi*, *Coxiella burnetii*, *Rickettsia* spp.) [228–232] and viruses (e.g., Crimean-Congo hemorrhagic fever virus) [233]. In Iran so far 16 species of the Ixodidae (hard ticks) from five genera (*Ixodes*, *Haemaphysalis*, *Rhipicephalus*, *Dermacentor* and *Hyalomma*) have been collected from horses, donkeys and mules (Table 5) [92, 96, 97, 233–244]. No species of the Argasidae (soft ticks) have been reported. As in older literature *Hyalomma excavatum* and *Hyalomma turanicum* were mentioned as subspecies of *Hyalomma anatolicum* and *Hyalomma marginatum*, we use the currently accepted name according to the most recent list of valid species names [245] although it is difficult to know exactly which species has been tested. The most commonly collected ticks from horses, donkeys and mules in different geographical regions of Iran are *H. anatolicum*, *Rhipicephalus bursa* and *Rhipicephalus annulatus* (Table 5).

**Table 5.** Tick species collected from horses, donkeys and mules in Iran

Tick species	Host	References
*Ixodes ricinus* (Linnaeus, 1758)	H	[240]
	D	[238]
*Haemaphysalis concinna* (Koch, 1844)	Not stated	[234]
*Haemaphysalis kopetdaghica* (Kerbabaev, 1962)	H	[244]
*Haemaphysalis punctata* (Canestrini & Fanzago, 1878)	H	[97, 240, 243]
	D	[97, 238]
*Rhipicephalus annulatus* (Say, 1821) (also reported as *Boophilus annulatus*)	H	[234–240]
	D	[236]
*Rhipicephalus bursa* (Canestrini & Fanzago, 1878)	H	[96, 97, 237, 238, 241, 243]
	D	[97, 242]
*Rhipicephalus sanguineus* (Latreille, 1806)	H	[92, 236, 237, 241]
*Dermacentor marginatus* (Sulzer, 1776)	H	[240, 243]
	D	[242]
*Hyalomma anatolicum* (Koch, 1844) (also reported as *Hyalomma anatolicum anatolicum*)	H	[96, 97, 234–237, 240, 241, 243]
	D	[97, 236, 238, 242]
	M	[238]
*Hyalomma asiaticum* (Schulze & Schlottke, 1930) (also reported as *Hyalomma asiaticum asiaticum*)	H	[237, 238]
*Hyalomma excavatum* (Koch, 1844) (also reported as *Hyalomma anatolicum excavatum*)	H	[92, 96, 235]
*Hyalomma detritum* (Schulze, 1919)	H	[236]
	D	[236]
	M	[236]
*Hyalomma dromedarii* (Koch, 1844)	H	[236]
	D	[236]
*Hyalomma impeltatum* (Schulze & Schlottke, 1930)	H	[236]
	D	[236]
*Hyalomma marginatum* (Koch, 1844) (also reported as *Hyalomma marginatum marginatum*)	H	H: [96, 237, 238, 241]
	D	[242]
*Hyalomma turanicum* (Pomerantsev, 1946) (also reported as *Hyalomma marginatum turanicum*)	H	[96, 236]
	D	[236]
	M	[236]

*H* horse, *D* donkey, *M* mule

At least 33 ixodid species in the genera *Hyalomma*, *Dermacentor*, *Rhipicephalus*, *Ixodes*, *Amblyomma* and *Haemaphysalis* have been implicated as competent vectors for *B. caballi*, *T. equi* or both [84]. In the only study on molecular detection of piroplasms in ticks infesting horses in Iran, the salivary glands of *H. excavatum* and *R. bursa* scored positive for *T. equi* in PCR, but no tick contained *B. caballi* DNA [96]. There is no other published research on examination of pathogens in ticks from equines in the country, although, for instance, *H. anatolicum*, *H. marginatum*, *R. bursa*, *R. sanguineus*, *H. asiaticum* and *H. dromedarii* are the most frequent species of tick vectors for Crimean-Congo hemorrhagic fever (CCHF) virus in Iran [246]. Horses and donkeys are known to be susceptible to CCHF virus although there is no evidence that they develop any symptomatic disease [233]. More studies are needed to define the role of equines in the epidemiology of tick-borne diseases.

### Mange mite infection

A variety of mites may infest equids. *Sarcoptes scabiei* var. *equi* (scabies and head mange), *Chorioptes bovis* (pastern mange), *Psoroptes equi* (body mange), *Pyemotes tritici* (straw itch mite) and *Trombicula* and *Eutrombicula* species (chiggers) are associated with pruritic equine skin diseases [247]. The prevalence of sarcoptic and psoroptic mange is very low among equines in Iran [248]. To date, *Psoroptes equi* [reported as *Psoroptes communis* var. *equi* (Hering)], *Sarcoptes scabiei* var. *equi* (Gerlach) and *Chorioptes bovis* have been isolated from horses in the country [249, 250].

### Lice infestation

Two types of lice feed on equines. Chewing lice (*Werneckiella equi* and *Bovicola* (*Werneckiella*) *ocellatus*) feed on the epidermal debris and prefer the dorsolateral trunk while sucking lice (*Haematopinus asini*) feed on blood and tissue fluid and most commonly infest the mane, tail and fetlock region [247, 251]. In Iran, infestations with *Haematopinus asini* and *Werneckiella equi* (reported as *Bovicola equi*) among horses and donkeys are rarely seen in the southeast and northeast areas [249, 252].

### Bot flies (*Gasterophilus* spp.)

The genus *Gasterophilus* (Oestridae, Gasterophilinae), known commonly as bot flies, include nine valid species [253]. Female flies attach eggs on the hair coat of equid hosts and larvae migrate to the oral cavity via different routes depending on the species. First-instar larvae reside in the mouth, but second- and third-instar larvae are found attached to the mucosa of different regions of the equid gastrointestinal tract, i.e., stomach, duodenum, colon or rectum. Generically, gasterophilosis is characterized by difficulties in swallowing (throat localization of the immature stages), gastric and intestinal ulcerations, gut obstructions or volvulus, rectal prolapse, anemia, diarrhea and digestive disorders [254]. In Iran four species of *Gasterophilus* (*G. intestinalis*, *G. nasalis*, *G. pecorum* and *G. inermis*) have been isolated from horses, donkeys, mules and Persian onagers [131, 158, 255–258]. Endoscopic examinations have shown that bot flies are causes of gastric ulcers in horses in northwestern Iran [259].

### Equine ked infestation

*Hippobosca equina*, also known as “forest fly,” usually parasitizes horses but also bites cattle, dogs, red deer, camelids, rabbits and humans. Adult winged flies lay larvae in the environment, where they immediately pupate, and new winged adults hatch from pupae, starting host-seeking behavior [260]. Humans bitten by this ked species often require emergency treatment because of allergic reactions [261]. These keds are regarded as both mechanical and biological vectors of *Corynebacterium pseudotuberculosis* [262]. Although *H. equina* is known to occur in Iran [263], so far infestation of horses and mules has been reported from the south of the country [264].

## Archeoparasitological findings

Since 2010 an international team has been studying the Iranian salt mine of Chehrabad, in the province of Zanjan, which was in operation under the Achaemenids (sixth to fourth century BC) and Sassanids (fourth to sixth century AD). Archeologists discovered the mummified remains of five miners who had been killed in a mining accident, and since then an extensive excavation has been initiated. Other than parasites of the mummies, *Oxyuris equi* eggs have been found in coprolites of equines in the site [265]. Furthermore, eggs of *F. hepatica* in coprolites of equids dating back to 224–651 AD [266], *Anoplocephala* spp. and strongyles eggs in equine coprolites [267] show that these parasites have been present in equids in Iran from ancient times.

## Conclusions

The present review reflects the current state of knowledge on the parasitic fauna of equids in Iran. Parasites and parasitic diseases of equines have not received adequate attention compared with those of ruminants and camels. Regarding helminths, as horse meat is not consumed in Iran because of cultural and religious beliefs there are no slaughterhouse data. Furthermore, although donkeys were slaughtered and fed to zoo carnivores in the last decades and their parasite fauna could be evaluated, this practice stopped almost 10 years ago upon the Glanders outbreak in tigers and lions in Tehran Zoo [268]. Hence, there is a need for country-wide planning of careful examination of a limited number of horses and donkeys that are killed for educational purposes or die for various reasons. A collaboration among parasitologists, pathologists and field veterinarians will make this goal achievable. Infection of equids with eyeworms also has not received adequate attention although the two species *T. lacrymalis* and *T. rhodesi* are endemic in some parts of the country [189, 190]. Moreover, several outbreaks of trichinellosis were associated with consumption of horse meat [269] but there is no information on equines in Iran. Detection of protozoan infections has been focused mainly on serological studies of *T. gondii* and *N. caninum*. Further research is needed based on multilocus PCR-RFLP genotyping [270] to improve current understanding of the transmission dynamics of infected equines to people consuming their milk. Identification and genotyping of *Cryptosporidium* spp. and *Giardia duodenalis* as zoonotic hazards have been neglected. A possible presence of *N. hughesi*, *Sarcocystis* (*S. neurona*, *S. bertrami* and *S. fayeri*) [271] and potentially zoonotic *Blastocytis* [272] and microsporidia [273] in the country requires further investigations. Regarding ticks, our information about the presence of pathogens in ixodids is also limited to a single study while *T. equi* and *B. caballi* have been reported from almost all regions of the country. In the absence of anthelmintics for horses in the market in Iran, there is a lack of products labeled for use in horses with known pharmacokinetics and pharmacodynamics as well as safety levels. Extra-labeled products, e.g., fenbendazole suspensions and ivermectin solutions formulated for ruminants and mebendazole formulated for humans, are commonly administered to horses. Hence, examination of the efficacy of formulations specific for horses with benzimidazoles, tetrahydropyrimidines, macrocyclic lactones, piperazine and praziquantel is essential [274]. The gap in the production of horse wormers should be filled by domestic pharmaceutical companies.

## Supplementary information

**Additional file 1: Text S1.** Persian translation of the abstract.

## Data Availability

All data generated or analysed during this study are included in this published article.

## Abbreviations

PCR: Polymerase chain reaction
DNA: Deoxyribonucleic acid
CCHF: Crimean-Congo hemorrhagic fever
DT: Dye test
MAT: Modified agglutination test
IFAT: Immunofluorescent antibody test
LAT: Latex agglutination test
