# Supplementary material for: Parasitic diseases of equids in Iran (1931–2020): a literature review
Source: Parasit Vectors. 2020 Nov 19;13:586. doi: 10.1186/s13071-020-04472-w (PMC7676409; doi:10.1186/s13071-020-04472-w)
Supplement: Supplementary file 1 — Additional file 1: Text S1. Persian translation of the abstract. [file 13071_2020_4472_MOESM1_ESM.pdf]

چکیده

آلودگی‌های انگلی می‌توانند باعث ایجاد بسیاری از بیماری‌های تنفسی، گوارشی یا سایر بیماری‌ها شوند و در برخی تغییرات عملکردی در تک‌سمی‌ها نقش دارند. با این حال، آگاهی درباره تنوع انگل‌های آن‌ها نیز هنوز محدود است. بررسی حاضر کلیه‌ی اطلاعات مربوط به بیماری‌های انگلی اسب‌ها، الاغ‌ها، قاطرها و الاغ‌های وحشی در ایران را که از سال ۱۹۳۱ تا ژوئیه ۲۰۲۰ به عنوان مقالات ژورنالی در مجلات ایرانی و بین المللی، پایان‌نامه‌ها و مقالات کنگره‌ای منتشر شده است را شامل می‌شود. انگل‌هایی که تاکنون از تکسمی‌های ایران توصیف شده است شامل ۹ جنس از تک‌یاخته‌ها (*تریپانوزوما*، *ژیاردیا*، *ایمریا*، *کلوسیلا*، *کریپتوسپوریدیوم*، *توکسوپلاسما*، *نئوسپورا*، *تیلریا* و *بابزیا*)، ۵۰ گونه‌ی کرم از دستگاه گوارش (یعنی، ۲ گونه از ترماتودها، ۳ گونه از سستودها و ۳۷ گونه از نماتودها)، و از سایر اندام‌ها (یعنی، *شیستوزوما تورکستانیکا*، *اکینوکوکوس گرانولوزوس*، *دیکتیوکاولوس آرنفیلدی*، *پارافیلاریا مولتی‌پاپیلوزا*، *ستاریا اکوئینا* و سه گونه *اونکوسرکا*) می‌باشند. به علاوه، ۱۶ گونه از کنه‌های سخت، ۳ گونه جرب عامل گری، ۲ گونه شپش، ۴ چهار گونه *گاستروفیلوس* و *هیپوبوسکا اکوئینا* از تک‌سمی‌های ایران گزارش شده است. یافته‌های باستان‌انگل‌شناسی در کوپرولایت‌های تک‌سمی‌ها شامل *فاسیولا هپاتیکا*، *اوکسیوریس اکوئی*، گونه‌ای از *آنوپلوسفالا* و استرونگل‌ها می‌باشند. بیماری-های انگلی از نظر رفاه حیوانات، اقتصاد و بهداشت عمومی موضوعات مهمی هستند؛ با این حال، به انگل‌ها و بیماری‌های انگلی تک‌سمی‌ها در مقایسه با نشخوارکنندگان و شترها در ایران به اندازه کافی توجه نشده است. مرور حاضر بر شکاف دانش در مورد وجود، گونه‌ها، ژنوتیپ‌ها و زیرگروه‌های *نئوسپورا هوقسیای*، گونه‌های *سارکوسیستیس*، گونه‌های *تریشینلا*، گونه‌های *کریتپوسپوریدیم*، *ژیاردیا دوئودنالیس*، *بلاستوسیستیس* و میکروسپوریدیا درتک‌سمی‌ها تاکید می‌کند. شناسایی کنه‌های ناقل انگل‌ها، باکتری‌ها و ویروس‌های بیماری‌زا نیز به طور اندک مورد توجه قرار گرفته است. کارایی کرم‌کش‌های معمول نیز می‌بایستی به طور سیستماتیک مورد ارزیابی قرار گیرد.
